# The first evaluation of the in vitro effects of silver(I)-*N*-heterocyclic carbene complexes on *Encephalitozoon intestinalis* and *Leishmania major* promastigotes

**DOI:** 10.1007/s00775-024-02063-z

**Published:** 2024-06-26

**Authors:** Ahmet Duran Ataş, Zübeyda Akın-Polat, Derya Gül Gülpınar, Neslihan Şahin

**Affiliations:** 1 Departments of Parasitology, Faculty of Medicine, Cumhuriyet University, 58140 Sivas, Turkey; 2 Department of Science Education, Faculty of Education, Cumhuriyet University, 58040 Sivas, Turkey

**Keywords:** *Encephalitozoon intestinalis*, *Microsporidia*, *Leishmania*, Silver, *N*-heterocyclic carbene

## Abstract

*Encephalitozoon intestinalis* is an opportunistic microsporidian parasite that primarily infects immunocompromised individuals, such as those with HIV/AIDS or undergoing organ transplantation. Leishmaniasis is responsible for parasitic infections, particularly in developing countries. The disease has not been effectively controlled due to the lack of an effective vaccine and affordable treatment options. Current treatment options for *E. intestinalis* infection and leishmaniasis are limited and often associated with adverse side effects. There is no previous study in the literature on the antimicrosporidial activities of Ag(I)-*N*-heterocyclic carbene compounds. In this study, the in vitro antimicrosporidial activities of previously synthesized Ag(I)-*N*-heterocyclic carbene complexes were evaluated using *E. intestinalis* spores cultured in human renal epithelial cell lines (HEK-293). Inhibition of microsporidian replication was determined by spore counting. In addition, the effects of the compounds on *Leishmania major* promastigotes were assessed by measuring metabolic activity or cell viability using a tetrazolium reaction. Statistical analysis was performed to determine significant differences between treated and control groups. Our results showed that the growth of *E. intestinalis* and *L. major* promastigotes was inhibited by the tested compounds in a concentration-dependent manner. A significant decrease in parasite viability was observed at the highest concentrations. These results suggest that the compounds have potential anti-microsporidial and anti-leishmanial activity. Further research is required to elucidate the underlying mechanisms of action and to evaluate the efficacy of the compounds in animal models or clinical trials.

**Graphical abstract:**

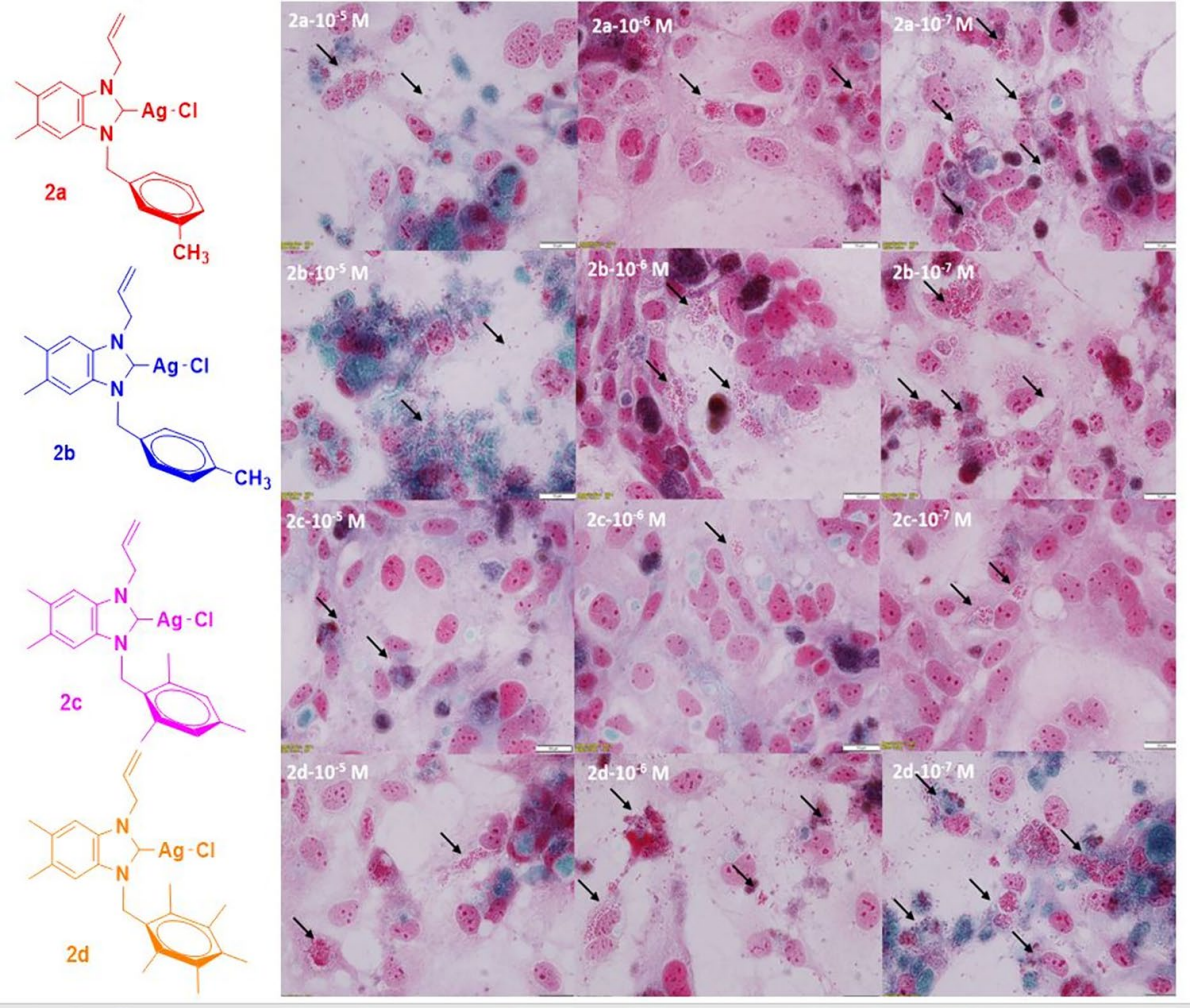

## Introduction

**Fig. 1:**
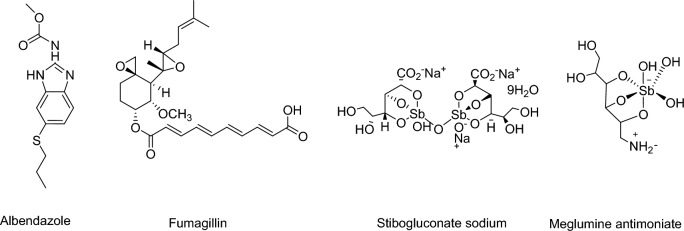
Treatment drugs for *Leishmaniasis* and *Microsporidia* infections

The genus *Encephalitozoon* includes protozoan parasites that are classified under the Microsporidia phylum. These obligate intracellular organisms cause a wide range of clinical symptoms, particularly in individuals with compromised immune systems, leading to significant morbidity and mortality. Despite the use of antiretroviral therapy, which is not fully effective and less accessible in developing countries where advanced HIV infection is common, *Encephalitozoon* infections continue to pose challenges [1–3]. Besides affecting individuals with AIDS, these microsporidia are increasingly reported in organ transplant recipients, children, travelers, contact lens wearers, and the elderly [2–6]. Intestinal microsporidiosis typically presents with persistent diarrhea, malabsorption, abdominal pain, and weight loss, while disseminated infections can lead to symptoms such as conjunctivitis, sinusitis, myositis, pneumonia, peritonitis, hepatitis, or nephritis [2, 7–9]. Treatment of Microsporidia infections is a challenging process due to the unique characteristics and intracellular nature of these parasites. Antimicrobial agents form the basis of the treatment approach for *Microsporidia* infections. Drugs such as albendazole and fumagillin can be effective by targeting the replication and growth of the parasite (Fig. 1). However, some *Microsporidia* species may develop resistance to drugs and fail to respond to treatment, leading to treatment failures [2, 10, 11]

Leishmaniasis is a neglected disease caused by protozoan parasites of the genus *Leishmania* transmitted by the bite of infected sandflies. The disease manifests in four different clinical forms, namely visceral leishmaniasis (VL or kala-azar), post-kala-azar dermal leishmaniasis (PCLD), cutaneous leishmaniasis (CL) and mucocutaneous leishmaniasis (CML) [12, 13]. As per the World Health Organization (WHO) reports, each year, more than one million new infections attributed to cutaneous leishmaniasis (CL) are documented in both developed and developing countries [14, 15]. Numerous drugs and treatment protocols are available for the treatment of cutaneous leishmaniasis (CL), including local and intralesional therapies, systemic therapy and alternative treatments. The World Health Organization (WHO) recommends local treatments, which include cryotherapy, thermotherapy (e.g. laser, infrared light and direct electrical stimulation), topical (e.g. paromomycin) and intralesional drug therapies [16]. Systemic therapy includes the use of antimonials, antifungals, antidepressants, amiodarone and immunomodulators. Among these, stibogluconate sodium and meglumine antimoniate, which are the primary antimonials, are highly effective and are considered the first-line treatment for leishmaniasis (Fig. 1) [16, 17]. Both intralesional and systemic antimonials are recommended by the WHO and the Pan American Health Organization (PAHO) for the treatment of CL. However, despite their recommendation, certain drugs prescribed for *Leishmania* infections have disadvantages such as lower sensitivity, non-specificity, toxicity, high cost, long-term therapeutic regimens, drug and parasite resistance, painful routes of administration, treatment failure and tissue damage. Consequently, there is a need to explore alternative drugs or therapeutic compounds for the treatment of CL [17–19].

*N*-heterocyclic carbenes (NHCs) are the most studied building block compounds of organometallic chemistry. Due to *N*-heterocyclic carbenes having strong σ-donating and weak π-accepting properties, they provide great stability for metal compounds [20–22]. In addition to their catalytic properties, it has been discovered in recent years that they also show high biological activity. Compounds of *N*-Heterocyclic carbenes with metals such as silver, gold and rhodium are studied intensively in medicinal chemistry [23–30].

In the class of metal-NHCs, Ag-NHCs have attracted attention because of their biological properties such as antibacterial activity, anticancer activity and antioxidant activity. Due to, the high stability of Ag-NHC complexes and slowly release of silver ions to the diseased area from Ag-NHC complexes, the Ag-NHC complexes show remerkable biological activity [31, 32]. For example, some silver-NHC complexes are used in the treatment of cystic fibrosis [33]. Haque et al. reported that the mono- and binuclear silver-NHC complexes showed high activity against E. coli and S. aureus bacterial strains in many studies [34–36]. In a 2021 study, picolyl and benzyl linked biphenyl NHC-silver complexes were found very active antibacterial agents [37]. A study conducted to examine the antiviral activities of silver-NHC complexes showed that silver NHC complexes represent a promising novel type of SARS-CoV-2 PLpro inhibitors [38]. In the study by Castro et al., it was stated that macrocyclic silver-NHC complexes could inhibit virus infectivity by interacting with HIV-1 envelope proteins [39].

When the literature was reviewed, it was seen that no study was conducted on antimicrosporidial activities of Ag(I)-NHC compounds. The primary objective of this study was to identify specific compounds with potential as new and more effective therapeutic agents against *E. intestinalis* and *L. major* promastigotes. These parasites are known to present challenges in terms of treatment efficacy. The study focused on investigating the effects of these compounds on *E. intestinalis* and *L. major* promastigotes through in vitro experiments. By studying the effects of these compounds on the parasites in a controlled laboratory environment, the study aimed to contribute to the development of improved treatment strategies for these infections.

## Materials and methods

### General procedure for the preparation of Ag(I)-NHC complexes

Ag(I)-NHC complexes (**2a–d**) were prepared by using standard Schlenk techniques under an inert argon atmosphere according to the previously our studies [40, 41]. The benzimidazolium salts synthesized according to the literature (**1a–d**) (1 mmol) and silver(I)oxide (2 mmol) were stirred in dichloromethane (25 mL) at room temperature for 24 h wrapped in aluminum foil. The solution obtained by filtration on celite was concentrated to 5 mL under vacuum. To resulting solution was added diethyl ether. The precipitated product was filtered and washed with diethyl ether. The yellow-gray solid was dried under vacuum and Ag-NHC complexes (**2a–d**) were obtained.

#### Chloro[1-allyl-3-(3-methylbenzyl)-5,6-dimethylbenzimidazole-2-ylidene]silver(I), 2a

Yield:74%; m.p. 152–153 °C, FT-IR *ν*_(CN)_: 1393 cm^−1^, LC–MS: 689.3 [AgL_2_]^+^. ^1^H NMR (400 MHz, CDCl_3_) δ (ppm): 2.31 (s, 3H, CH_2_C_6_H_4_-C*H*_3_-3), 2.31 (s, 3H, NC_6_H_2_N(C*H*_3_)_2_–5,6) 2.36 (s, 3H, NC_6_H_2_N(C*H*_3_)_2_–5,6), 5.01 (d, 2H, NC*H*_2_CHCH_2_, *J* = 8 Hz), 5.20 (d, 1H, NCH_2_CHC*H*_2_, *J* = 12 Hz), 5.32 (d, 1H, NCH_2_CHC*H*_2_, *J* = 8 Hz), 5.50 (s, 2H, C*H*_2_C_6_H_4_-CH_3_-3), 6.01 (quint, 1H, NCH_2_C*H*CH_2_, *J* = 4 Hz), 7.01–7.27 (m, 6H, Ar–*H*). ^13^C{^1^H} NMR (100 MHz, CDCl_3_) δ (ppm): 20.4 (NC_6_H_2_N(*C*H_3_)_2_–5,6), 21.4 (CH_2_C_6_H_4_-*C*H_3_-3), 51.9 (*C*H_2_C_6_H_4_-CH_3_-3), 53.3 (N*C*H_2_CHCH_2_), 124.1 (NCH_2_CH*C*H_2_), 132.0 (NCH_2_*C*HCH_2_), 112.0, 112.2, 119.0, 127.7, 128.9, 129.2, 132.4, 132.5, 133.8, 135.0, 138.9 (Ar–*C*), no peak (*C*_carbene_-Ag). Anal. Calcd. for C_20_H_22_AgClN_2_: C: 55.38 H: 5.11; N: 6.46. Found C: 55.99; H: 5.50; N: 6.36.

#### Chloro[1-allyl-3-(4-methylbenzyl)-5,6-dimethylbenzimidazole-2-ylidene]silver(I), 2b

Yield: 76%; m.p. 180–181 °C, FT-IR *ν*_(CN)_: 1393 cm^−1^, LC–MS: 689.3 [AgL_2_]^+^. ^1^H NMR (400 MHz, CDCl_3_) δ (ppm): 2.31 (s, 6H, NC_6_H_2_N(C*H*_3_)_2_–5,6), 2.35 (s, 3H, CH_2_C_6_H_4_-C*H*_3_-4), 5.00 (d, 2H, NC*H*_2_CHCH_2_, *J* = 8 Hz), 5.19 (d, 1H, NCH_2_CHC*H*_2_, *J* = 16 Hz), 5.31 (d, 1H, NCH_2_CHC*H*_2_, *J* = 8 Hz), 5.51 (s, 2H, C*H*_2_C_6_H_4_-CH_3_-4), 6.00 (quint, 1H, NCH_2_C*H*CH_2_, *J* = 4 Hz), 7.11–7.19 (m, 6H, Ar–*H*). ^13^C{^1^H} NMR (100 MHz, CDCl_3_) δ (ppm): 20.4 (NC_6_H_2_N(*C*H_3_)_2_–5,6), 21.1 (CH_2_C_6_H_4_-*C*H_3_-4), 51.8 (*C*H_2_C_6_H_4_-CH_3_-4), 53.1 (N*C*H_2_CHCH_2_), 129.7 (NCH_2_CH*C*H_2_), 133.8 (NCH_2_*C*HCH_2_), 112.0, 112.2, 119.0, 127.1, 132.0, 132.1, 132.4, 132.5, 138.3 (Ar–*C*), no peak (*C*_carbene_-Ag). Anal. Calcd. for C_20_H_22_AgClN_2_: C: 55.38 H: 5.11; N: 6.46. Found C: 56.20; H: 5.62; N: 6.39.

#### Chloro[1-allyl-3-(2,4,6-trimethylbenzyl)-5,6-dimethylbenzimidazole-2-ylidene]silver(I), 2c

Yield: 77%; m.p. 228–229 °C; FT-IR *ν*_(CN)_: 1393 cm^−1^, LC–MS: 743.4 [AgL_2_]^+^. ^1^H NMR (400 MHz, CDCl_3_) δ (ppm): 2.23 (s, 6H, NC_6_H_2_N(C*H*_3_)_2_–5,6), 2.35 (s, 6H, CH_2_C_6_H_2_(C*H*_3_)_3_–2,4,6), 2.38 (s, 3H, CH_2_C_6_H_2_(C*H*_3_)_3_–2,4,6), 4.95 (d, 2H, NC*H*_2_CHCH_2,_* J* = 4 Hz) 5.12 (d, 1H, NCH_2_CHC*H*_2_, *J* = 20 Hz), 5.25 (d, 1H, NC*H*_2_CHC*H*_2_, *J* = 8 Hz), 5.40 (s, 2H, C*H*_2_C_6_H_2_(CH_3_)_3_–2,4,6), 5.93 (quint., 1H, NCH_2_C*H*CH_2_, *J* = 8 Hz), 6.99 (s, 2H, Ar–*H*), 7.09 (s, 1H, Ar–*H*), 7.19 (s, 1H, Ar–*H*). ^13^C{^1^H} NMR (100 MHz, CDCl_3_) δ (ppm): 20.3 (NC_6_H_2_N(*C*H_3_)_2_–5,6), 20.4 (CH_2_C_6_H_2_(*C*H_3_)_3_–2,4,6), 20.5 (CH_2_C_6_H_2_(*C*H_3_)_3_–2,4,6), 21.2 (CH_2_C_6_H_2_(*C*H_3_)_3_–2,4,6), 47.2 (*C*H_2_C_6_H_2_(CH_3_)_3_–2,4,6), 52.4 (N*C*H_2_CHCH_2_), 118.6 (NCH_2_CH*C*H_2_), 130.3 (NCH_2_*C*HCH_2_), 111.7, 112.0, 126.7, 132.0, 132.3, 133.0, 133.5, 133.8, 137.5, 139.6 (Ar–*C*), no peak (*C*_carbene_-Ag). Anal. Calcd. for C_22_H_26_AgClN_2_ × 0.5H_2_O: C: 56.13 H: 5.78; N: 5.95. Found C: 56.62; H: 5.75; N: 6.05.

#### Chloro[1-allyl-3-(2,3,4,5,6-pentamethylbenzyl)-5,6-dimethylbenzimidazole-2-ylidene]silver(I), 2d

Yield: 79%; m.p. 232–233 °C, FT-IR *ν*_(CN)_: 1393 cm^−1^, LC–MS:801.4 [AgL_2_]^+^. ^1^H NMR (400 MHz, CDCl_3_) δ (ppm): 2.19 (s, 6H, NC_6_H_2_N(C*H*_3_)_2_–5,6), 2.28 (s, 6H, CH_2_C_6_(C*H*_3_)_5_–2,3,4,5,6), 2.33 (s, 3H, CH_2_C_6_(C*H*_3_)_5_–2,3,4,5,6), 2.40 (s, 3H, CH_2_C_6_(C*H*_3_)_5_–2,3,4,5,6), 2.41 (s, 3H, CH_2_C_6_(C*H*_3_)_5_–2,3,4,5,6), 4.92 (d, 2H, NC*H*_2_CHCH_2_, *J* = 4 Hz), 5.10 (d, 1H, NCH_2_CHC*H*_2_, *J* = 16 Hz), 5.23 (d, 1H, NCH_2_CHC*H*_2_, *J* = 12 Hz), 5.39 (s, 2H, (C*H*_2_C_6_(CH_3_)_5_–2,3,4,5,6), 5.91 (quint, 1H, NCH_2_C*H*CH_2_, *J* = 4 Hz), 7.19 (s, 1H, Ar–*H*), 7.30 (s, 1H, Ar–*H*), 10.61 (s, 1H, NC*H*N). ^13^C{^1^H} NMR (100 MHz, CDCl_3_) δ (ppm): 17.1 (NC_6_H_2_N(*C*H_3_)_2_–5,6), 17.2, (CH_2_C_6_(*C*H_3_)_5_–2,3,4,5,6), 17.4 (CH_2_C_6_(*C*H_3_)_5_–2,3,4,5,6), 20.4 (CH_2_C_6_(*C*H_3_)_5_–2,3,4,5,6), 20.5 (CH_2_C_6_(*C*H_3_)_5_–2,3,4,5,6), 47.3 (*C*H_2_C_6_(CH_3_)_5_–2,3,4,5,6), 52.7 (N*C*H_2_CHCH_2_), 126.7 (NCH_2_CH*C*H_2_), 134.2 (NCH_2_*C*HCH_2_), 111.5, 112.1, 118.8, 132.2, 133.0, 133.2, 133.4, 133.8, 137.3 (Ar–*C*), no peak (*C*_carbene_-Ag). Anal. Calcd. for C_24_H_30_AgClN_2_ × 0.5H_2_O: C: 57.79 H: 6.26; N: 5.62. Found C: 58.00; H: 5.76; N: 5.86.

### Cytotoxic potential of Ag(I)-NHC complexes

#### Cultivation of human kidney epithelial cells (HEK-293)

HEK-293 (human kidney epithelial cells, ATCC) cells were used in the study. Cells were grown in DMEM medium containing 10% fetal bovine serum (FBS), 1% penicillin–streptomycin solution (Thermo-Fisher Scientific, USA). Cultures were incubated under humid conditions in an oven at 37 °C with 5% CO2. Passages were made throughout the study to ensure continuity and proliferation of the HEK-293 cell line.

#### Cytotoxicity assay

Cells were seeded into 96-well microtiter plates (Costar, Corning, USA) at a concentration of 1 × 10^5^ cells/mL, with a final volume of 100 µL per well. The cells were then incubated in a humidified atmosphere at 37 °C and 5% CO_2_ for 24 h. All compounds were initially prepared as a stock solution in dimethylsulfoxide DMSO at a concentration of 10^−2^ M. To evaluate the cytotoxicity of the compounds, four different concentrations (10^−4^ M, 10^−5^ M, 10^−6^ M and 10^−7^ M) were prepared from the stock solution. The compounds were added to the cells at the prepared concentration and incubated for 24 h. After the incubation period, 10 µL of XTT labelling reagent was added to each well. After a 2-h tetrazolium reaction, the absorbance of the samples was measured at 450 nm using a microtiter plate reader (Thermo Scientific Microplate Photometer, Multiskan FC, USA), with the control consisting of the same cells without the tested compound. To establish a background control, the same volume of culture medium and XTT labelling reagent (10 µL XTT labelling reagent per 100 µL culture medium) was added to a separate well. In the experiments, DMSO was used as a solvent control in the same ratio as the DMSO containing the compounds. Cytotoxicity measurements were performed in four replicates. The optical density (OD) of the samples was compared with that of the negative control to determine the percentage viability using the formula: cell viability (%) = [(OD450 (sample)/OD450 (negative control)) × 100].

### In vitro antimicrosporidial activities

#### Parasites and culture

The reference strain of *E. intestinalis*, ATCC® 50,506™, was used in the study. The spores of *E. intestinalis* were cultured in vitro using human renal epithelial (HEK-293) cell lines. The cells were maintained in RPMI-1640 medium (SIGMA-Aldrich—St. Louis, MO) supplemented with 2.5% heat-inactivated fetal bovine serum. Spores were isolated and purified by centrifugation at 1100 g for 30 min. The spores were then washed three times with sterilized phosphate-buffered saline (PBS).

#### In vitro assay

HEK-293 cells were seeded on 48-well tissue culture plates at a density of 5 × 10^5^ cells per well. The cells were cultured in RPMI 1640 medium containing 2 mM L-glutamine and 5% fetal bovine serum at 37 °C in a 5% CO_2_ environment overnight to allow the host cells to reach confluence. The tissue culture plates were then infected by replacing the medium with fresh medium containing 10^5^ *E. intestinalis* spores/mL, resulting in a ratio of 5 parasites to 1 cell. The next day, the plates were washed with phosphate-buffered saline (PBS) to remove any spores that had not penetrated the cells. The three highest non-cytotoxic concentrations of the compounds were then added to the cells. On days 3 and 7, fresh medium containing the drug or diluent was added to each well without disturbing the parasites or cells. Controls were treated with proportionally diluted DMSO, which was used for the initial dissolution of the drugs. Drugs were added to specific wells in the tetraplate.

On day 10, a solution of 10% (wt/vol) sodium dodecyl sulphate (SDS) was added to all wells to release the microsporidia from the host cells. The number of microsporidia was then counted using a haemcytometer. Each treatment was performed in quadruplicate and the percentage inhibition of microsporidian replication was calculated using the formula 100—[(number of microsporidia in each treated well / mean number of microsporidia in untreated wells) × 100].

To visualise the microsporidial spores inside and outside the cell, sterile coverslips were placed on the bottom of 6-well tissue culture flasks in triplicate for each compound and the above procedures were repeated. On day 10, the coverslips were removed from the bottom of the flask and stained with Trichrom stain. The preparations were examined at X100 magnification and photographed.

### In vitro effects of *Leishmania major* promastigotes

#### Parasites and culture

The *L. major* strain (MHOM/TR/2013/MANISAPB145) was obtained from the parasite bank of Celal Bayar University and was grown mainly on NNN medium. When it reached the lagarithmic phase, it was inoculated into RPMI-1640 medium containing 10% fetal calf serum.

#### In vitro assay

Seeding of 100 µL of logarithmic growth phase promastigotes (5 × 10^5^ promastigotes/mL) into 96-well plates containing 100 µL of RPMI-1640 plus 10% FBS was performed considering the concentrations of Ag(I)-NHC complexes (**2a–d)** (10^−5^, 10^−6^ and 10^−7^ M) in the form of maintained at 24 ± 2 °C for 48 h. After the incubation period, 10 µL of XTT labelling reagent was added to each well of the microtitre plate. After a 2-h tetrazolium reaction, the absorbance of the samples was measured at 450 nm using a microplate reader (Thermo Scientific Microplate Photometer, Multiskan FC, USA). Absorbance values were compared with those of the control to assess the metabolic activity or cell viability of the samples. Promastigotes without drug and medium without parasites were used as positive and negative controls, respectively. Medium containing 2% DMSO was also used as a control. All experiments were carried out in tetraplicate.

### Statistical analysis

The collected data, which were revised and verified, were analysed using the Statistical Package for the Social Sciences (SPSS) program, specifically version 22.0 for Windows. The results were presented as mean ± standard deviation (SD) and were obtained from six replicates. Statistical analysis was performed using one-way analysis of variance (ANOVA) with a 95% confidence level for multiple comparisons. In addition, Student's t-test was used for comparisons between the two groups. The standard deviation (SD) provides information on the amount of variation or dispersion observed from the average or expected value (mean).

## Results and discussion

### Synthesis and characterization of Ag(I)–NHC complexes

**Scheme 1:**
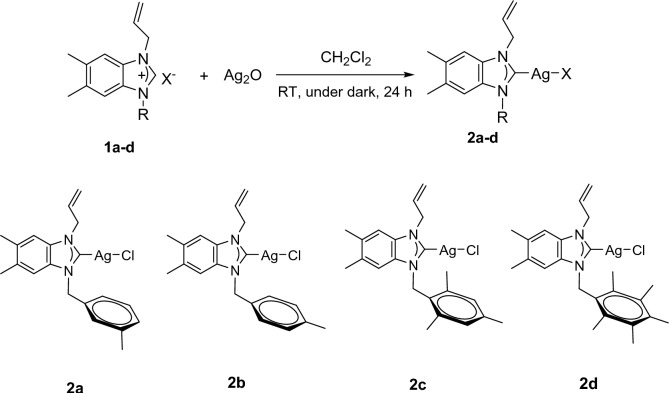
Synthesis of Ag(I)-NHC complexes, **2a–d**

Ag(I)-NHC complexes (**2a–d**) were synthesized according to our previous studies studies [40, 41]. To obtain Ag(I)-NHC complexes (**2a–d**), the benzimidazoluim salts (**1a–d**) with Ag_2_O were reacted in dichloromethane for 24 h and in good yields. The synthetic route was shown in Scheme 1.

The Ag(I)-NHC complexes (**2a–d**) were stable to air and moisture in solid and solution sensitive to light (Figure S1). The structure of the Ag(I)-NHC complexes (**2a–d**) was described previously by FT-IR, ^1^H, and ^13^C{^1^H} NMR spectroscopy and elemental analysis was also performed to evaluate the purity of the complexes.

NMR spectra of the complexes were analyzed in d-CDCl_3_. In ^1^H NMR spectra of Ag(I)-NHC complexes (**2a–d**), the protons of the methyl groups in the benzyl ring and benzimidazole ring gave signals around 1.0–2.0 ppm as singlets. Protons of the allyl were seen around 4.0–5.0 ppm, benzylic protons gave signals between 5.40 and 5.60 ppm as singlet and aromatic protons were seen around 7.10–7.60 ppm.

In ^13^C{^1^H} NMR spectra of Ag(I)-NHC complexes (**2a–d**), N*C*N carbon peaks were not seen. Because silver-NHC complexes are static or have dynamic behavior, carbene coupling to silver has been seen as slow on the NMR time scale. As the dynamic behavior increases on the NMR time scale, the carbene resonance will become a sharp singlet. In the opposite case, carbene resonance can not be seen. These evaluations are in agreement with reported data for similar compounds [42]. That’s why sometimes Ag-C_carbene_ peak is seen and sometimes not. Benzylic carbons were seen around 40.0–50.0 ppm, and aromatic carbons were seen around 120.0–150.0 ppm.

At the infrared spectra of the Ag(I)-NHC complexes **2a–d**, a specific υ_(C=N)_ band was observed 1393 cm^−1^ for all complexes. ^1^H and ^13^C{^1^H} NMR spectrum of compounds, and print out of elemental analysis are presented in detail in the Supplementary Files (Figure S2–S6).

LC–MS spectrometry of the prepared Ag(I)-NHC complexes showed maximal peak intensities for each complex assigned to [Ag(NHC)_2_]^+^ as the molecular ion in solution, which is not uncommon for this type of complexes. Fluxional behavior between [(NHC)AgX] and [(NHC)_2_Ag]^+^[AgX_2_]^−^ species was observed in solution and the mechanism of formation of complexes was reported. The carbenic carbon provides a weaker donation to the silver and indicates a weaker Ag-C bond. This weaker bond increases the lability of the carbene moieties, and provides the formation of [(NHC)_2_Ag]^+^and equilibrium between [(NHC)AgX] and [(NHC)_2_Ag]^+^ in solution [41].

### Cytotoxic potential of Ag(I)-NHC complexes

**Fig. 2:**
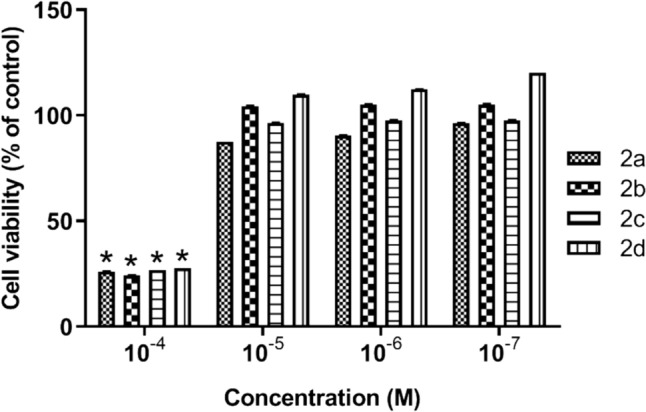
In vitro cytotoxicity of 10^−4^, 10^−5^, 10^−6^, and 10^−7^ M concentrations of Ag(I)-NHC complexes (**2a–d**) on human renal epithelial cells (HEK-293) by XTT method. *p < 0.05 vs. control

The initial evaluation of newly synthesised compounds for drug potential typically involves assessing their cytotoxicity to ensure they are safe for further development. Cytotoxicity assays provide critical data on the effects of compounds on cell viability, helping to identify and eliminate those with undesirable toxic profiles early in the drug discovery process. This step is fundamental in guiding subsequent in vitro and in vivo studies. According to our cytotoxicity results for this purpose, 10^−5^ M, 10^−6^ M and 10^−7^ M concentrations of **2a–d** complexes showed no toxic effect on HEK-293 cells and the difference was not statistically significant (p > 0.05). However, when the viability rate at a concentration of 10^−4^ M of the compounds was compared with the control, the difference was statistically significant (p < 0.05), indicating cytotoxicity (Fig. 2). As a result, concentrations of 10^−5^ M, 10^−6^ M, and 10^−7^ M, which did not show toxic effects on cells, were selected for further evaluation of their antimicrosporidial and anti-leishmanial activity.

### Antimicrosporidial and antileishmanial activity

**Fig. 3:**
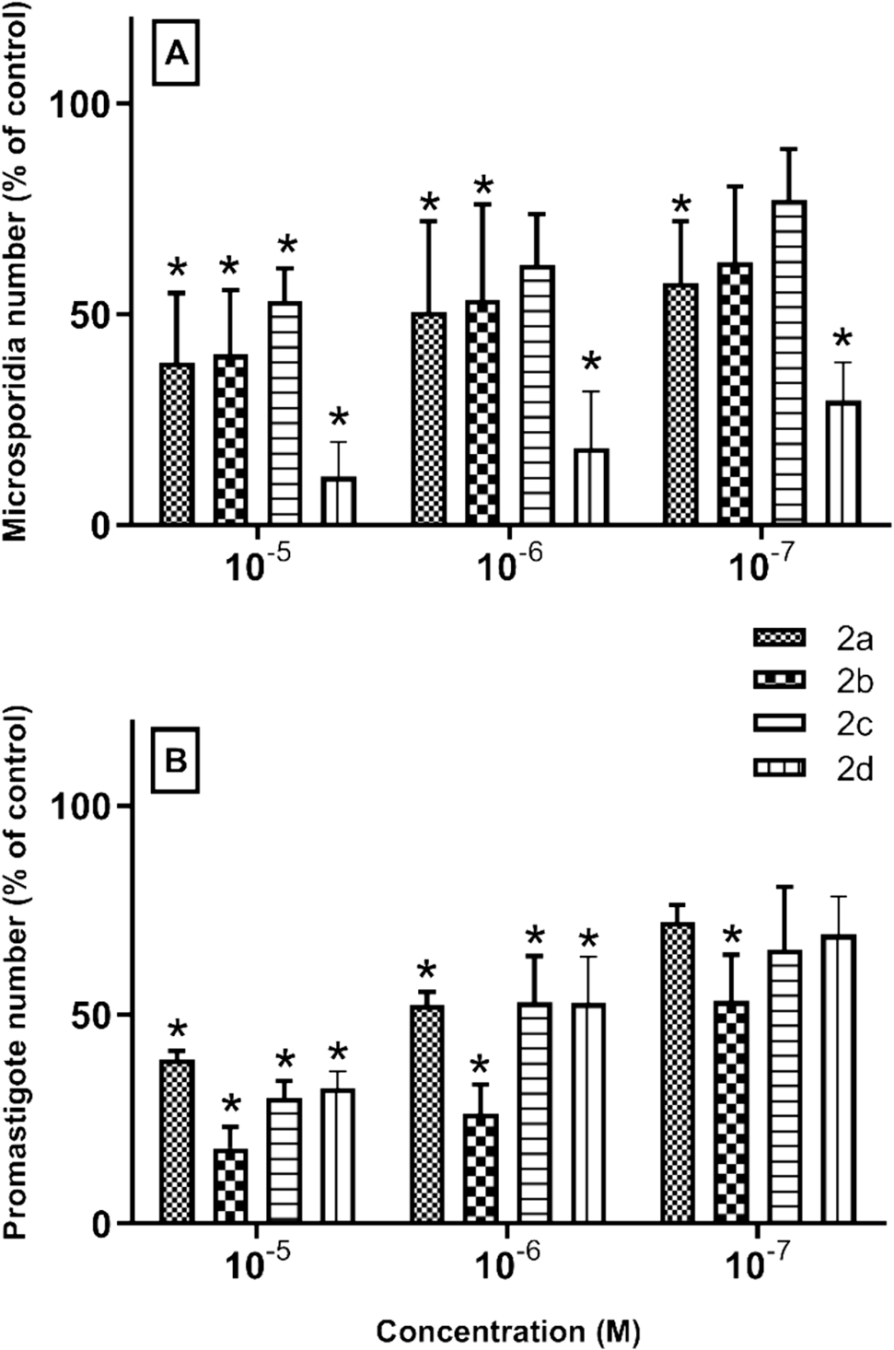
In vitro activity of 10^−5^, 10^−6^ and 10^−7^ M concentrations of Ag(I)-NHC complexes (**2a–d**) against *E. intestinalis* spores (**A**) and *L. major* promastigotes (**B**)*.* *p < 0.05 vs control. Data are expressed as mean ± SD

Microsporidiasis primarily affects immunocompromised individuals, with symptoms including chronic diarrhoea, wasting, and systemic infection. Treatment is hampered by the limited efficacy of available antiparasitic drugs, the emergence of drug resistance, and the lack of standardised therapeutic protocols [1, 8, 9]. Leishmaniasis manifests in three main clinical forms: cutaneous, mucocutaneous and visceral, the latter being the most severe and potentially fatal if untreated. Treatment is complicated by factors such as drug resistance, toxicity and the need for long and complex treatment regimens. In addition, access to effective treatment is limited in many endemic regions due to socio-economic constraints and health infrastructure deficits [14, 16, 17]. The results of our study showed that all tested compounds exhibited dose-dependent inhibitory effects on the proliferation of both *L. major* (a parasite that causes leishmaniasis) promastigotes and *E. intestinalis* (a microsporidian that causes microsporidiosis) spores compared to the control group (Table 1, Fig. 3). These results highlight the potential of these newly synthesized compounds as valuable tools in the development of novel therapeutic strategies against leishmaniasis and microsporidiosis.

**Table 1:** Antimicroporidial and anti-leishmanial activity of Ag(I)-NHC complexes (**2a–d**) in vitro

Compounds	Number of microsporidia			Absorbance value of the promastigotes		
Concentration	10^−5^ M	10^−6^ M	10^−7^ M	10^−5^ M	10^−6^ M	10^−7^ M
2a	145.5 ± 16.46	190.75 ± 21.54	216.5 ± 14.70	0659 ± 0.035	0.877 ± 0.045	1.209 ± 0.085
2b	152.5 ± 15.30	201.25 ± 22.76	235.25 ± 17.96	0.303 ± 0.015	0.441 ± 0.061	0.893 ± 0.035
2c	200.5 ± 7.63	233 ± 11.93	290.75 ± 12.07	0.506 ± 0.088	0.887 ± 0.068	1.098 ± 0.020
2d	43.5 ± 8.20	69.25 ± 13.25	111.25 ± 9.14	0.544 ± 0.094	0.885 ± 0.042	1.158 ± 0.050
Control	386.75 ± 16.61			1.473 ± 0.045		

Data were described using mean ± SD

Our study found that the new compounds we tested could slow down or stop the growth of both *L. major* (a parasite that causes leishmaniasis) and *E. intestinalis* (a microsporidian that causes microsporidiosis). The effects were stronger when we used higher doses of the compounds. These findings suggest that these new compounds might be useful for creating new treatments for these diseases.

Remarkably, complex **2d** showed remarkable efficacy in suppressing microsporidian proliferation, demonstrating its robust inhibitory effect on *E. intestinalis.* At concentrations of 10^−5^ M, 10^−6^ M, and 10^−7^ M, the inhibition rates against *E. intestinalis* spores were 88.46%, 81.62% and 70.48% respectively, in marked contrast to the control group, all with statistical significance (p < 0.01) (Fig. 3A).

**Fig. 4:**
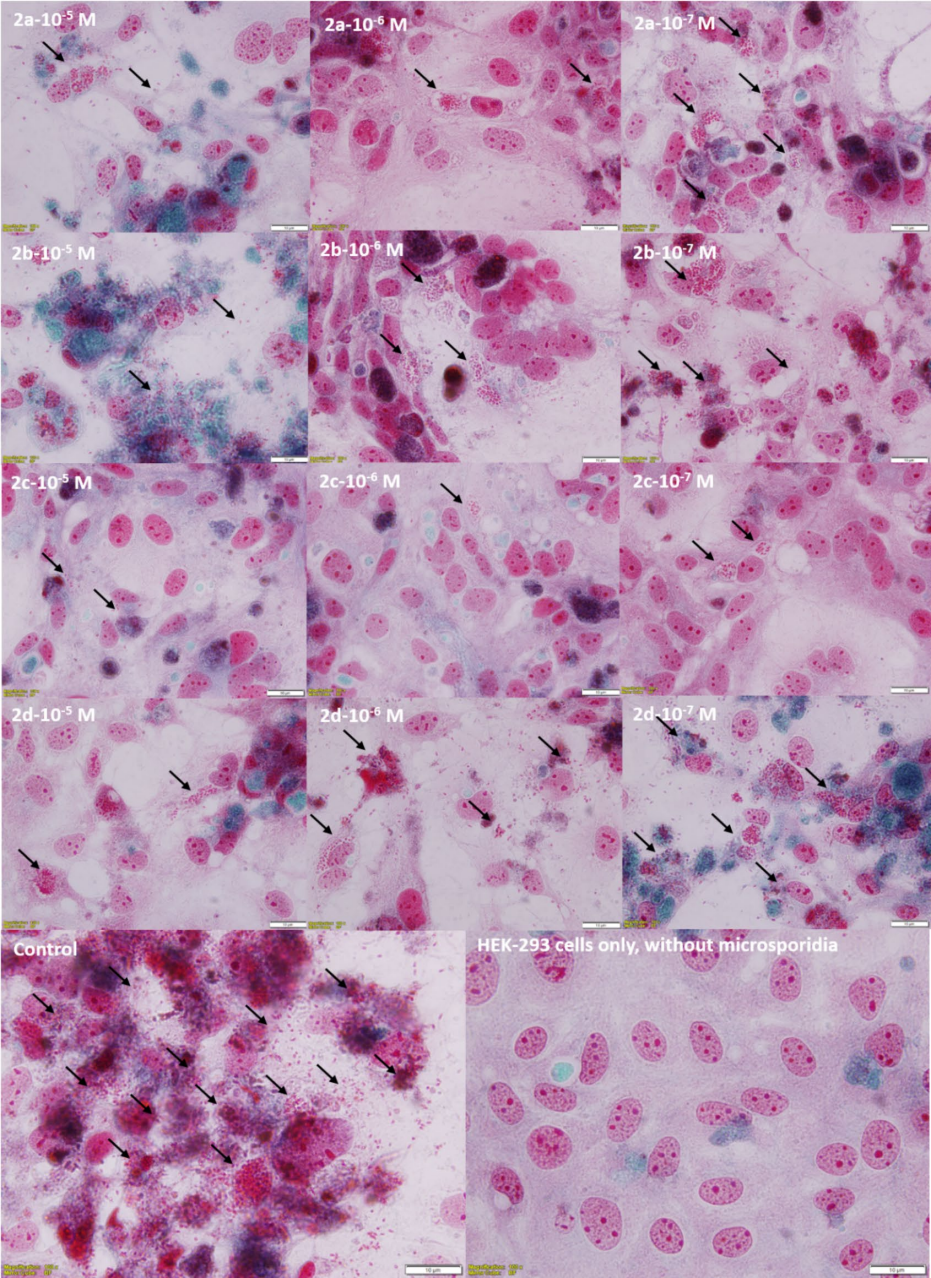
On day 10, the appearance of host cells and *E. intestinalis* spores were treated with three different concentrations of the Ag(I)–NHC complexes (**2a–d**) and stained with Trichrom stain (× 100). Spores are seen dispersed intracellularly and extracellularly (arrows)

Proliferation in HEK-293 cells treated with three different concentrations of the complexes was assessed by Trichrom staining at the end of the ten-day experimental period. The results were consistent with the experimental findings and showed that the density of E. intestinalis spores was low at all concentrations of all compounds. The density of *E. intestinalis* was lowest in complex **2d** and highest in complex **2c** (Fig. 4). This observation is particularly noteworthy as microsporidial infections pose significant challenges in clinical management due to limited treatment options and adverse effects in immunocompromised individuals [10, 11].

The potent inhibitory activity of **2d** suggests its potential as a lead complex for the development of therapeutics specifically targeting microsporidia. Future investigations should focus on elucidating the underlying mechanisms by which complex **2d** exerts its inhibitory effects on microsporidia, which may lead to the identification of novel molecular targets for intervention.

Complex **2b**, which was most effective against *L. major* promastigotes, inhibited promastigote proliferation at 10^−5^ M concentration by 81.89%, 10^−6^ M concentration by 73.65% and 10^−7^ M concentration by 46.63% compared to the control (p < 0.01) (Fig. 3B). The robust inhibitory activity of **2b** against *Leishmania* is promising considering the urgent need for new drugs to combat this neglected tropical disease. Further studies are warranted to elucidate the mechanisms underlying the efficacy of **2b** against *Leishmania*, which may lead to the development of novel therapeutic approaches against this parasite. The emergence of drug-resistant strains of *Leishmania* and the limitations of existing treatment options highlight the urgent need for the discovery and development of new therapeutic drugs for leishmaniasis [17–19]. The results of our study add to the growing body of evidence supporting the exploration of novel compounds as potential treatment options for this debilitating disease.

## Conclusion

In conclusion, our study highlights the dose-dependent inhibitory effects of four newly synthesized Ag(I)-NHC complexes on both *Leishmania* promastigotes and *E. intestinalis* as the first evaluation in the literature. Furthermore, the observed dose-dependent inhibitory effects of the tested compounds on both *Leishmania* promastigotes and *E. intestinalis* spores indicate their broad-spectrum activity against different parasite species. This property holds great promise for the development of therapeutics that can effectively target multiple parasitic infections, simplifying treatment protocols and improving patient outcomes. These results support the potential of these compounds as promising candidates for the development of novel therapeutic interventions against leishmaniasis and microsporidiosis. Further research is required to elucidate the precise mechanisms of action and to evaluate the therapeutic efficacy of these compounds in preclinical and clinical settings.

## Data Availability

The data that supports the findings of this study are available within the article.
